# Acceleration of Anaerobic Cysteine Transformations to Sulfane Sulfur Consequent to **γ**-Glutamyl Transpeptidase Inhibition

**DOI:** 10.1100/2012/253724

**Published:** 2012-04-30

**Authors:** Inga Kwiecień, Małgorzata Iciek, Lidia Włodek

**Affiliations:** ^1^Chair and Department of Pharmaceutical Botany, Medical College, Jagiellonian University, Medyczna 9, 30-688 Kraków, Poland; ^2^Chair of Medical Biochemistry, Medical College, Jagiellonian University, Kopernika 7, 31-034 Kraków, Poland

## Abstract

Toxicity of drugs and radiation in the cells is largely dependent on the level of thiols. In the present studies, an attempt has been made to inhibit *γ*-glutamyl transpeptidase (*γ*GT) activity in EAT-bearing animals tissue. We have expected that administration of *γ*GT inhibitors: acivicin and 1,2,3,4-tetrahydroisoquinoline (TIQ) may influence GSH/*γ*–glutamyl transpeptidase (*γ*GT) system in the regulation of cysteine concentration and anaerobic cysteine metabolism in normal and cancer cells. Development of Ehrlich ascites tumor in mice enhances peroxidative processes, diminishes levels of nonprotein thiols (NPSH) and sulfane sulfur, and lowers activities of enzymes involved in its formation and transfer in the liver and kidney. Although *γ*GT inhibitors further decrease NPSH level, they increase cysteine and sulfane sulfur levels. This means that upon *γ*GT inhibition, cysteine can be efficiently acquired by normal liver and kidney cells via another pathway, that is so productive that sulfane sulfur level and intensity of anaerobic cysteine metabolism even rise.

## 1. Introduction

**Scheme 1:**
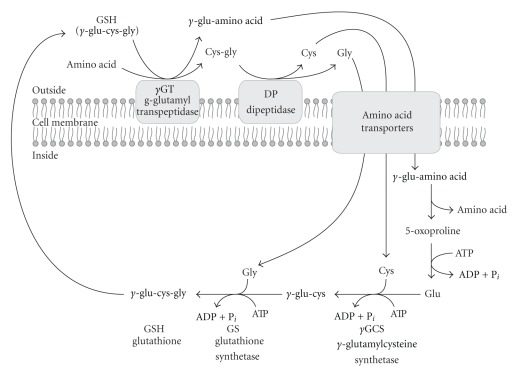
*γ*-Glutamyl cycle.

Biological functions of thiols in the cell depend on the activity of their hydrosulfide group, owning to which they can participate in antioxidant and detoxifying reactions [1]. Reduced glutathione (GSH) is responsible for maintaining proper thiol-disulfide balance and related cellular redox potential. GSH shows the ability to regenerate protein –SH groups, thereby preventing oxidative damage and inactivation of proteins. It is involved in detoxification of hydrogen peroxide and organic peroxides via glutathione peroxidase pathway. GSH can also react nonenzymatically with hydroxyl radical (^•^OH), and with ONOO^−^, O_2_, and O_2_^•−^ [2]. Under physiological conditions, glutathione (GSH) and cysteine concentrations in cytosol and in extracellular space are regulated by efficiently functioning Meister's *γ*-glutamyl cycle (Scheme 1) [3]. In that process, GSH is translocated outside the cell, where it is hydrolyzed to compound amino acids by transmembrane peptidases: *γ*-glutamyl transpeptidase and cysteinylglycine dipeptidase. GSH cannot be transported inside the majority of cells; therefore, extracellular hydrolysis catalyzed by *γ*-glutamyl transpeptidase enables cysteine return and GSH resynthesis in the cell. This process fulfils also a function of amino acid transport, protection, and modulation of membrane redox potential. Maintenance of the physiological cysteine level in the cell and extracellular space is also one of important roles of the *γ*-glutamyl cycle. It means that cysteine availability in the cell is contingent not only on methionine biodegradation rate and cysteine/cystine transport mechanisms but also on the availability of this amino acid derived from extracellular GSH hydrolysis.

**Scheme 2:**
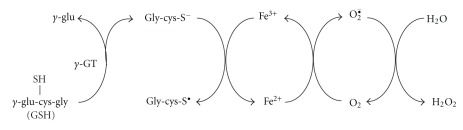
Hydrogen peroxide generation in the reaction catalyzed by *γ*-glutamyl transpeptidase (*γ*GT).

In the last decade, an increasing body of evidence has indicated that reactive oxygen species-generating reactions can play a significant role in the regulation of the most important processes in the cell, like proliferation, apoptosis, cell adhesion, and gene expression [4]. GSH was shown to have antioxidant properties, associated with its extracellular hydrolysis to an exceptionally active thiol, cysteinylglycine (Cys-Gly) (Scheme 1). This thiol dipeptide is characterized by lower pK_a_ of –SH group than GSH, what facilitates reactive oxygen species generation during reduction of metal cations (Fe^3+^) [5] (Scheme 2). Since *γ*-glutamyl transpeptidase is indispensable for GSH resynthesis in the cell, it is considered to be an antioxidant enzyme, but it can be classified as a prooxidant enzyme, either, due to the product of GSH hydrolysis, Cys-Gly. For this reason, inhibition of *γ*-glutamyl transpeptidase activity can lead to both cysteine deficit in the cell and consequent decline in GSH level, and to the elimination of a physiological source of oxidative stress.

**Scheme 3:**
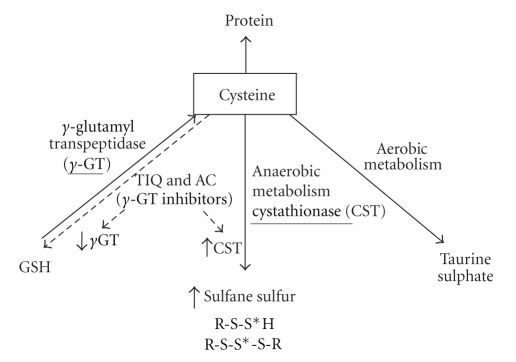
Pathways of cysteine metabolism and putative effects of *γ*GT inhibition.

 Cysteine supplied with diet or formed during methionine trasnsulfuration is incorporated into proteins and GSH besides, it can be metabolized *via *aerobic pathway to sulfates and taurine and *via* anaerobic pathway to a pool of sulfane sulfur-containing compounds (Scheme 3).

 Sulfane sulfur occurs in the 0 or −1 oxidation state and is always bound to another sulfur atom. Sulfane sulfur is generated during anaerobic cysteine metabolism with *γ*-cystathionase as a key enzyme (Scheme 3) [6–8]. Cystathionase substrate is a disulfide, cystine, while its products contain sulfane sulfur, that is, unstable cysteine hydropersulfide Cys-S-S*-H (thiocysteine) and stable trisulfide, thiocystine Cys-S-S*-S-Cys (S*-sulfane sulfur). Both these compounds are substrates of rhodanese, which catalyzes reaction of sulfane sulfur transfer to different acceptors, thereby fulfilling its biological role (Scheme 3) [9]. Sulfane sulfur atom is characterized by exceptional reactivity and easiness of leaving the structure of parent compound, being transferred to different acceptors. These enzymes can also transport sulfane sulfur in the form of hydropersulfides and trisulfides [10, 11]. Rhodanese function principally consists in sulfane sulfur transfer from anionic donors (thiosulfate, persulfides, polysulfides, polythionates) to thiophilic acceptors (cyanide, sulfate IV) yielding thiocyanate and thiosulfate, respectively, and to iron-sulfur proteins [12]. The reduced sulfane sulfur shows also a powerful reductive capacity. Therefore, anaerobic cysteine metabolism results in covalent modification of –SH groups to form unstable protein hydropersulfides and trisulfides, whereby thiols produce their regulatory and antioxidant effects. Thiocysteine, that is, cysteine hydropersulfide is characterized by greater ability to donate protons and electrons and, therefore, by stronger antioxidant properties in comparison with the cysteine –SH groups. Moreover, perthiyl radicals formed concomitantly (RSS^•^) are more stable and, thus, more dangerous than thiyl radicals (RS^•^) [13].

 One of significant differences between normal and cancer cells is much higher content of sulfane sulfur and greater activities of enzymes involved in its formation and transport in the former [14]. Therefore, taking into account: (1) different rate of anaerobic cysteine metabolism in normal and cancer cells and (2) role of GSH/*γ*-glutamyl transpeptidase system in the regulation of cysteine concentration, we decided to investigate the effect of *γ*-glutamyl transpeptidase inhibitors on anaerobic sulfur metabolism. The studies involved the application of a known *γ*-glutamyl transpeptidase inhibitor, acivicin [15], and 1,2,3,4-tetrahydroisoquinoline, whose capacity to inhibit *γ*-glutamyl transpeptidase was observed in our previous studies [16]. Next, the effect of these inhibitors on sulfane sulfur level, activities of enzymes participating in its metabolism and transfer, and on intensity of peroxidative processes was determined in the liver and kidney of Ehrlich ascites tumor-bearing mice and in Ehrlich ascites tumor cells.

The aim of the present studies was to precisely identify the differences in the activity and role of *γ*-glutamyl transpeptidase (*γ*GT) and *γ*-cystathionase (*γ*CT) in thiols metabolism between normal and tumor cells. This selectivity can supposedly be used in order to increase efficacy and safety of anticancer therapies.

## 2. Materials and Methods

### 2.1. Animals

Male Swiss albino mice, weighing approximately 20 g, were randomly divided into four groups. Intraperitoneal inoculation of Ehrlich ascites tumor cells was performed to animals (group 2–4), and the mice were maintained for 5 days. Chemicals dissolved in isotonic saline were i.p. injected at the following doses: acivicin at 18 mg per kg of body weight (group 3) and 1,2,3,4-tetrahydroisoquinoline at 50 mg per kg of body weight (group 4). Control mice (group 1 and 2) were injected with the same volume of 0.9% sodium chloride solution. On the fifth day, the mice were sacrificed. The Ehrlich ascites tumor cells were collected and washed three times by suspension into cold 0.9% sodium chloride solution, followed by centrifugation at 650 ×g for 5 min. The livers and kidneys were isolated, placed in liquid nitrogen, and stored at −70°C until biochemical tests were performed. Homogenates of the tissues were prepared by homogenization of 1 g of tissue in 4 mL of 0.1 M phosphate buffer pH 7.4 at a temperature of 4°C.

All procedures were approved by the Ethics Committee for the Animal Research in Kraków (nr 74/OP/2002).

### 2.2. Reagents

Acivicin, 2′,7′-dichlorohydrofluorescein (DCHF-DA), 5,5′dithiobis-2-nitrobenzoic acid (DTNB), dithiothreitol, homoserine, L-*γ*-glutamyl-p-nitroanilide, glycylglycine, lactic dehydrogenase, 3-mercaptopyruvate, 3-methyl-2-benzo-thiazolinone hydrazone (MBTH), NADH, potassium cyanide, pyridoxal 5′-phosphate (PLP), p-phenylenediamine, 1,2,3,4-tetrahydroisoquinoline, and trichloroacetic acid (TCA) were obtained from Sigma Chemical Company, St. Louis, (USA). The remaining chemicals were purchased from POCh, Gliwice, (Poland).

### 2.3. Methods

#### 2.3.1. Determination of Nonprotein Sulfhydryl Groups [17]

In this paper, 5,5′-dithiobis-2-nitrobenzoic acid is reduced by nonprotein sulfhydryl groups present in TCA extract to 2-nitro-5-mercaptobenzoic acid. For the estimation of nonprotein sulfhydryl groups, 0.05 mL of TCA extract and 0.1 mL of 6 mM DTBN were added in succession to 0.850 mL of 0.2 M phosphate buffer pH 8.2, and absorbance was measured at 412 nm.

#### 2.3.2. Determination of Free Cysteine [18]

The levels of reduced form of cysteine were assayed by colorimetric reaction with ninhydrin solution. To 0.95 mL of homogenate, 0.05 mL of 50% TCA was added and centrifuged at 12500 ×g for 10 min. Subsequently, to 0.125 mL of supernatant, there were added 0.125 mL of 5% TCA, 0.125 mL of 99.5% acetic acid and 0.125 mL of ninhydrin reagent (250 mg of ninhydrin, 6 mL of acetic acid, and 4 mL of hydrohloric acid). The reaction mixture was incubated in a boiling-water bath for 10 min, cooled, and then 0.5 mL of ethanol was added. Absorbance was measured at 560 nm.

#### 2.3.3. Determination of Reactive Oxygen Species [19]

 2′7′-Dichlorohydrofluorescein diacetate (DCFH-DA) was deestrified in homogenates to dichlorohydrofluorescein and was then oxidized to fluorescent dichlorofluorescein by reactive oxygen species. To 1.2 mL of 0.1 M phosphate buffer, pH 7.4, 0.01 mL of homogenate and 0.01 mL of DCFH-DA were added. Mixtures were incubated in a water bath for 30 min at 37°C and then centrifuged at 12000 ×g for 8 min. Fluorescence was measured with an excitation of 488 nm and an emission of 525 nm.

#### 2.3.4. Determination of L-*γ*-Glutamyl Transpeptidase Activity [20]

In this method, the colourless substrate L-*γ*-glutamyl-p-nitroanilide was enzymatically converted to p-nitroaniline. The reaction mixture (0.9 mL) contained 5 mM L-*γ*-glutamyl-p-nitroanilide and 11 mM of MgCl_2_ dissolved in 111 mM Tris-HCl buffer, pH 9 and was incubated for 5 min at 37°C with 50 *μ*L of tissue homogenates. Adding 1 mL of 1.5 M acetic acid stopped the reaction. The probes were centrifuged at 12000 ×g for 5 min, and the absorbance of p-nitroaniline that developed during 5 min was measured at 410 nm.

#### 2.3.5. Determination of Sulfane Sulfur Level

The level of the sulfur in tissues homogenate was determined by cold cyanosis according to Wood [21]. To 0.1 mL of tissues homogenate 0.08 mL of 1 M NH_3_, 0.72 mL distilled water and 0.1 mL of 0.5 M KCN were added. The samples were incubated at room temperature for 45 min. Then 0.02 mL of 38% formaldehyde and 0.2 mL of Goldstein's reagent (Fe(NO)_3_ + HNO_3_ + H_2_O) were added. After centrifugation at 12000 ×g for 10 min, the absorbance at 460 nm was determined.

The pool of sulfane sulfur in Ehrlich ascites tumor cells was assayed by the modification of Ogasawara's method [22]. In this method, bound sulfur is easily liberated as sulfide after reduction by dithiotreitol. The released sulfide is converted into a fluorescent derivate, thionine, in the reaction with p-phenylenediamine and ferric ion. Thionine is determined by fluorimetric method.

#### 2.3.6. Determination of Rhodanese Activity [23]

Enzymatic activity of rhodanese was determined according to Sörbo. Assay mixture containing 100 ml of diluted homogenate, 60 mM thiosulfate, 47.5 mM sodium phosphate buffer pH 7.4, and 71.5 mM cyanide was incubated in final volume of 525 ml at room temperature for 5 min. The reaction was stopped by addition of formaldehyde. Reaction product, that is, thiocyanide and ferric ions, formed coloured complex, whose absorbance was measured spectrophotometrically at 460 nm.

#### 2.3.7. Determination of *γ*-Cystathionase Activity

Enzymatic activity of cystathionase was determined according to Soda [24] with modifications. The assay mixture contained 0.02 *μ*mol of PLP, 4 *μ*mol of homoserine, 0.065 mmol of potassium phosphate buffer, and tissues homogenate in final volume of 1 mL. The mixture was incubated at 37°C for 30 min, and the reaction was stopped by adding 50% TCA. After centrifugation (14000 ×g/10 min) to 0.5 mL supernatant 1 mL acetate buffer pH 8.0 and 0.4 mL of 0.1% MBTH were added. Incubation was carried out at 50°C for 30 min. Absorbance at 320 nm was measured after cooling the reaction mixture.

#### 2.3.8. Determination of 3-Mercaptopyruvate Sulfotransferase (MPST) Activity [25]

Determination of 3-mercaptopyruvate sulfotransferase (MPST) involves two steps. MPST catalyzes sulfur transfer from 3-mercaptopyruvate to sulfate (IV) yielding pyruvate and thiosulfate during 15 min incubation at 37°C. Next pyruvate is reduced to lactate by lactic dehydrogenase in the presence of NADH, which is oxidized to NAD^+^. This method utilizes a difference in absorbance of NADH and NAD^+^ at 340 nm, which is a measure of the amount of pyruvate formed in MPST-catalyzed reaction.

#### 2.3.9. Determination of Protein Content Using Lowry's Method [26]

### 2.4. Statistical Analysis

The results are presented as the means ± S.D., and statistical significance of differences was evaluated using ANOVA followed by at Least Significance test and Difference test for posthoc comparisons. The differences were considered statistically significant when *P* < 0.05.

## 3. Results

**Figure 1:**
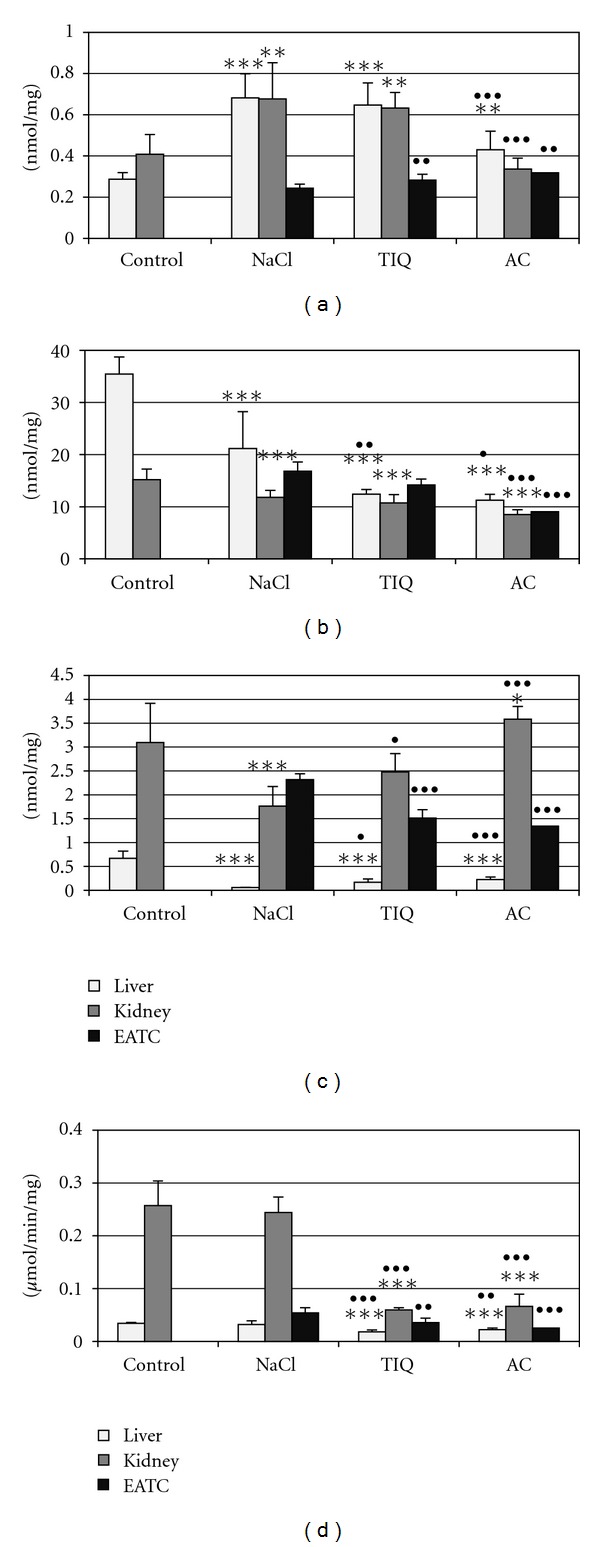
Reactive oxygen species (a), nonprotein sulfhydryl groups (b), cysteine (c) level, and *γ*-glutamyltranspeptidase activity (d) in healthy and Ehrlich ascites tumor- (EAT-) bearing mice livers, kidneys, and EAT cells. 0.9% NaCl (control groups), 1,2,3,4-tetrahydroisoquinoline (TIQ) at 50 mg·kg^−1^ of body weight, and acivicin (AC) at 18 mg·kg^−1^ were i.p. injected for 5 days. *-*P* < 0.05 versus control, **-*P* < 0.01 versus control, ***-*P* < 0.001 versus control, ^•^-*P* < 0.05 versus NaCl, ^••^-*P* < 0.01 versus NaCl, ^•••^-*P* < 0.001 versus NaCl.

**Figure 2:**
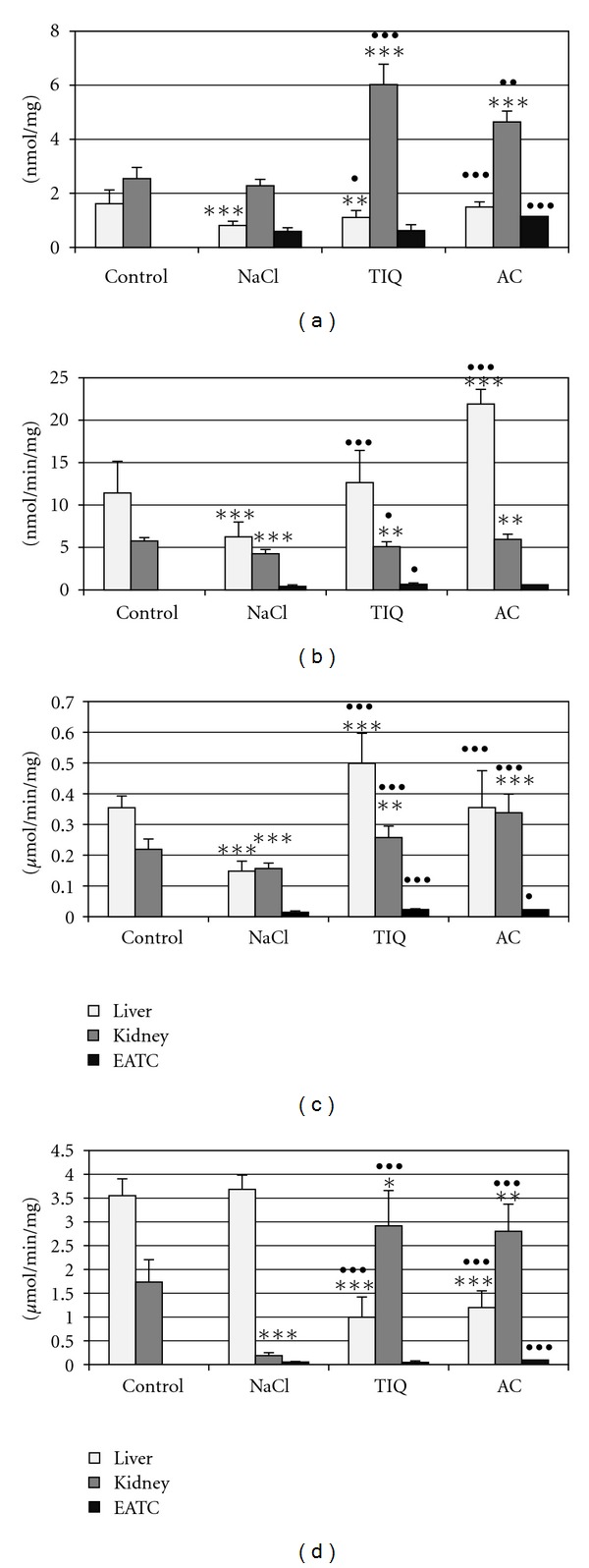
Sulfane sulfur level (a), cystathionase (b), rhodanese (c), and 3-mercaptopyruvate transferase activity (d) in healthy and Ehrlich ascites tumor- (EAT-) bearing mice livers, kidney, and EAT cells. 0.9% NaCl (control groups), 1,2,3,4-tetrahydroisoquinoline (TIQ) at 50 mg·kg^−1^ of body weight, and acivicin (AC) at 18 mg·kg^−1^ were i.p. injected for 5 days. *-*P* < 0.05 versus control, **-*P* < 0.01 versus control, ***-*P* < 0.001 versus control, ^•^-*P* < 0.05 versus NaCl, ^••^-*P* < 0.01 versus NaCl, ^•••^-*P* < 0.001 versus NaCl.

Developing tumor caused an increase in reactive oxygen species level (Figure 1(a)), which means that it accelerated peroxidative processes in the liver and kidney of Ehrlich ascites tumor-bearing mice in comparison with healthy animals. It was accompanied by a drop in nonprotein sulfhydryl groups, cysteine (Figures 1(b), and 1(c)), and sulfane sulfur level (Figure 2(a)). Activities of enzymes involved in sulfane sulfur biosynthesis and transport, like cystathionase and rhodanese, and 3-mercaptopyruvate sulfotransferase in the kidney (Figures 2(b), 2(c), and 2(d)) declined, while *γ*-glutamyl transpeptidase activity remained at the control level (Figure 1(d)).

*γ*-Glutamyl transpeptidase inhibitors, like 1,2,3,4-tetrahydroisoquinoline and acivicin, diminished activity of this enzyme in the liver and kidney of Ehrlich ascites tumor-bearing mice and in Ehrlich ascites tumor cells (Figure 1(d)). *γ*-Glutamyl transpeptidase inhibition by both inhibitors was accompanied by a statistically significant increase in sulfane sulfur (Figure 2(a)) and cysteine level (Figure 1(c)), a rise in cystathionase and rhodanese activity in the kidney and liver (Figures 2(b), and 2(c)), and a drop in reactive oxygen species and nonprotein thiol level (Figures 1(a) and 1(b)). Interestingly, hepatic activity of 3-mercaptopyruvate sulfotransferase declined after both inhibitors, while renal activity of this enzyme increased (Figure 2(d)).

On the other hand, in Ehrlich ascites tumor cells, both inhibitors (1,2,3,4-tetrahydroisoquinoline and acivicin) lowered nonprotein sulfhydryl groups level and cysteine concentration (Figures 1(b) and 1(c)), whereas sulfane sulfur level rose after acivicin, parallelly with elevation in cystathionase, rhodanese, and 3-mercaptopyruvate sulfotransferase activities (Figures 2(b), 2(c), and 2(d)).

*γ*-Glutamyl transpeptidase inhibitors differently affected peroxidative processes in the liver and kidney: acivicin lowered reactive oxygen species level in the liver and kidney while 1,2,3,4-tetrahydroisoquinoline did not change it (Figure 1(a)). On the other hand, both compounds statistically significantly elevated reactive oxygen species level in Ehrlich ascites tumor cells (Figure 1(a)).

## 4. Discussion

*γ*-Glutamyl transpeptidase is a membrane enzyme composed of a heavy subunit, anchored in the membrane and light subunit located on the membrane surface and containing the active center [27]. GSH hydrolysis to a dipeptide Cys-Gly is executed on the outer side of plasma membrane and is catalyzed by *γ*-glutamyl transpeptidase. The dipeptide is further hydrolyzed to cysteine and glycine by a dipeptidase (Scheme 1) [28].

Mice lacking *γ*-glutamyl transpeptidase activity in the kidney and liver have lower GSH level, which confirms the role of this enzyme in GSH biosynthesis [29]. Ramos B lymphoma exhibit signs of oxidative stress and apoptosis in cystine-free medium, which is another example corroborating this thesis, the more so that lymphoma cells transfected with *γ*-glutamyl transpeptidase do not undergo apoptosis under such conditions, due to their ability to synthesize GSH and acquire cysteine from extracellular GSH hydrolysis [30, 31]. Hence, a seemingly paradoxical situation can be observed when suppression of the GSH-degrading enzyme leads to the inhibition of its biosynthesis due to cysteine deficit in the cell.

The results presented in this paper also indicate that *γ*-glutamyl transpeptidase activity is responsible for cellular GSH level. *γ*-Glutamyl transpeptidase blockade by inhibitors (1,2,3,4-tetrahydroisoquinoline and acivicin) results in a drop in nonprotein thiol level (of which GSH constitutes 95%) in the kidney and liver of Ehrlich ascites tumor-bearing mice and in Ehrlich ascites tumor cells. Interestingly, our research also demonstrated that acivicin significantly diminished reactive oxygen species level in normal hepatic and renal cells from Ehrlich ascites tumor-bearing mice while 1,2,3,4-tetrahydroisoquinoline did not change it versus control level. Conversely, in Ehrlich ascites tumor cells both inhibitors increased reactive oxygen species level. This means that the *γ*-glutamyl transpeptidase inhibitors-induced decrease in nonprotein sulfhydryl groups level was accompanied by impairment of antioxidant capacity only in tumor cells. Based on these observations, it can be suggested that the action of *γ*-glutamyl transpeptidase inhibitors is selectively beneficial for normal cells.

 The obtained results indicated that *γ*-glutamyl transpeptidase inhibitors increased cysteine level in normal cells and decreased it in tumor cells, which was also confirmed by other authors [32].

 The increase in cysteine level in normal cells, induced by both inhibitors, was accompanied by a rise in cystathionase activity, implicated in sulfane sulfur biosynthesis, which explains the increased level of the sulfane sulfur, observed in our experiments. Augmentation of sulfane sulfur content, induced by both *γ*-glutamyl transpeptidase inhibitors in the liver and kidney and in Ehrlich ascites tumor cells, coexisted with significantly elevated rhodanese activity, which indicates that sulfane sulfur transfer to different acceptors was accelerated, as well. The most dramatic increase in cystathionase activity was seen in the liver (even above activity observed in healthy animals) while in the kidney, normal level was recovered. It is peculiar that hepatic activity of 3-mercaptopyruvate sulfotransferase, the second enzyme implicated in sulfane sulfur formation, decreased after *γ*-glutamyl transpeptidase inhibitors, whereas its activity in the kidney and Ehrlich ascites tumor cells increased.

 The drop in reactive oxygen species level, observed in our studies in normal cells of the kidney, and liver can be theoretically explained by suppression of oxidative stress associated with GSH/*γ*-glutamyl transpeptidase system activity; however, this was not paralleled by similar change in Ehrlich ascites tumor cells. Hence, the rise in antioxidant capacity in normal cells, particularly in the kidney, most probably can be consequent to both elevated concentration of cysteine and products of its metabolism to a pool of sulfane sulfur-containing compounds, characterized by a strong antioxidant potential.

 Therefore, we observed that *γ*-glutamyl transpeptidase inhibition lowered GSH level (due to the decreased nonprotein sulfhydryl groups), while cysteine and sulfane sulfur contents and cystathionase activity (in the kidney also 3-mercaptopyruvate sulfotransferase) rose. However, unexpectedly a drastic increase in cystathionase activity, the main enzyme of sulfane sulfur biosynthesis, was observed in the liver, although the highest sulfane sulfur level occurs in the kidney. Therefore, it can be supposed that sulfane sulfur formed in the liver in the reaction catalyzed by cystathionase is transported in the form of albumin hydropersulfides in the circulation and then is stored in the kidney. It cannot be excluded that to some extent renal sulfane sulfur level could also be affected by the increased 3-mercaptopyruvate sulfotransferase activity, which is involved in sulfane sulfur biosynthesis, too.

 It is interesting to note that hepatic and renal cysteine level, decreased due to developing tumor in Ehrlich ascites tumor-bearing mice, rose after *γ*-glutamyl transpeptidase inhibitors. It was particularly conspicuous in the kidney but also significant albeit smaller in the liver. The increase in cysteine concentration indicates that upon *γ*-glutamyl transpeptidase blockade, these organs are able to acquire this amino acid independently of *γ*-glutamyl cycle. Consequently, cysteine in normal cells can act as an antioxidant or can be metabolized via anaerobic pathway and increase sulfane sulfur pool.

 It has to be emphasized that the kidneys are characterized by the highest physiological level of cysteine [33] and sulfane sulfur [22] and *γ*-glutamyl transpeptidase activity [34]. It means that this organ plays an exceptional role in the metabolism of the most important thiols; however, significance of this fact has not been fully elucidated, yet.

Such situation was not observed in Ehrlich ascites tumor cells, namely, both inhibitors lowered cysteine level. Although concomitantly sulfane sulfur level and activity of related enzymes rose, the antioxidant capacity was limited due to their very low level.

In summary, development of Ehrlich ascites tumor in mice enhances peroxidative processes, diminishes levels of nonprotein thiols and sulfane sulfur, and lowers activities of enzymes involved in its formation and transfer in the liver and kidney. Although *γ*-glutamyl transpeptidase inhibitors further decrease nonprotein sulfhydryl groups level (of which GSH constitutes 95%), they increase cysteine and sulfane sulfur levels. This means that upon *γ*-glutamyl transpeptidase inhibition, cysteine can be efficiently acquired by normal liver and kidney cells via another pathway, that is so productive that sulfane sulfur level and intensity of anaerobic cysteine metabolism even rise. Hence, the concomitant decrease in reactive oxygen species level in normal cells can be consequent to both elevated cysteine and sulfane sulfur levels, which upon *γ*-glutamyl transpeptidase blockade will take over the antioxidant function compensating for GSH deficit. Therefore, *γ*-glutamyl transpeptidase inhibitors can selectively strengthen antioxidant defense (by increasing cysteine and sulfane sulfur levels) only in normal cells of Ehrlich ascites tumor-bearing animals.

On the contrary, in Ehrlich ascites tumor cells, *γ*-glutamyl transpeptidase inhibitors lower nonprotein thiol level, including cysteine, which was accompanied by acceleration of oxidative processes (the increase in reactive oxygen species). This indicates that Ehrlich ascites tumor cells are much more dependent on cysteine supply from GSH *via* *γ*-glutamyl transpeptidase pathway in *γ*-glutamyl cycle. Although sulfane sulfur level and activities of related enzymes in tumor cells increase either, it does not seem to play a significant antioxidant role due to very low sulfane sulfur concentrations.

This means that EAT cells, in contrast to normal liver and kidney cells, are completely dependent on cysteine supply from the reaction catalyzed by *γ*-glutamyl transpeptidase. Inhibition of *γ*-glutamyl transpeptidase activity significantly limited antioxidant defense as well as drug detoxification in this cancer cells and could selectively increase their vulnerability to chemo- and radiotherapy.
